# From Information Seekers to Innovators: Qualitative Analysis Describing Experiences of the Second Generation of E-Patients

**DOI:** 10.2196/13022

**Published:** 2019-08-15

**Authors:** Therese Scott Duncan, Sara Riggare, Sabine Koch, Lena Sharp, Maria Hägglund

**Affiliations:** 1 Health Informatics Centre Department of Learning, Informatics, Management and Ethics Karolinska Institutet Stockholm Sweden; 2 Division of Innovative Care Research Department of Learning, Informatics, Management and Ethics Karolinska Institutet Stockholm Sweden; 3 Regional Cancer Centre Stockholm - Gotland Stockholm County Council Stockholm Sweden; 4 Department of Womens and Childrens Health Uppsala University Uppsala Sweden

**Keywords:** consumer health informatics, eHealth, qualitative research, self-care, motivation

## Abstract

**Background:**

Current health care systems are rarely designed to meet the needs of people living with chronic conditions. However, some patients and informal caregivers are not waiting for the health care system to redesign itself. These individuals are sometimes referred to as e-patients. The first generation of e-patients used the internet for finding information and for communicating with peers. Compared with the first generation, the second generation of e-patients collects their own health data and appears to be more innovative.

**Objective:**

The aim of this study was to describe the second generation of e-patients through exploration of their active engagement in their self-care and health care.

**Methods:**

Semistructured interviews were conducted with 10 patients with chronic conditions and 5 informal caregivers. They were all recruited through a Web-based advertisement. Data were analyzed according to the framework analysis approach, using the 3 concepts of the self-determination theory—autonomy, relatedness, and competence—at the outset.

**Results:**

Study participants were actively engaged in influencing their self-care and the health care system to improve their own health, as well as the health of others. This occurred at different levels, such as using their own experience when giving presentations and lectures to health care professionals and medical students, working as professional peers in clinical settings, performing self-tracking, contributing with innovations, and being active on social media. When interaction with health care providers was perceived as being insufficient, the participants sought support through their peers, which showed strong relatedness. Competence increased through the use of technology and learning experiences with peers. Their autonomy was important but was sometimes described as involuntary and to give up was not an option for them.

**Conclusions:**

Like the first generation of e-patients, the participants frequently searched for Web-based information. However, the second generation of e-patients also produce their own health data, which they learn from and share. They also engage in the innovation of digital tools to meet health-related needs. Utilizing technological developments comes naturally to the second generation of e-patients, even if the health care system is not prepared to support them under these new circumstances.

## Introduction

### Background

Many patients, especially those with chronic or long-term conditions, and their informal caregivers experience a need to be actively involved in care provision to co-ordinate contacts with health care professionals and navigate the health care system [1]. Unfortunately, health care systems today are rarely designed to meet the current needs. However, some patients and informal caregivers are not waiting for the health care system to redesign itself. They take matters into their own hands and create innovative approaches and solutions to manage their care and interactions with health care providers, often with the use of electronic health (eHealth) solutions [2]. These individuals are sometimes referred to as e-patients, and in this study conducted in Sweden, we explored how their activities have evolved.

### First Generation of E-Patients—Information Seekers

The first generation of e-patients was described by Ferguson and Frydman [3] as citizens who use the internet for searching for health information or use electronic communication tools to solve a personal health-related need. The concept of e-patients includes both patients and informal caregivers who receive *better health information and services, and different (but not always better) relationships with their doctors* [3]. The term *informal caregiver* is used in this study to represent a family member or other person, for example, a close friend, who supports and cares for a patient without being formally employed or reimbursed to do so. The *e* in e-patients primarily stands for *electronic;* however other, more descriptive attributes are mentioned, describing these digitally literate patients and informal caregivers as equipped, empowered, enabled, and engaged in their self-care and in health care [4]. We will use this concept of e-patients in this study, which includes both patients and informal caregivers who have these descriptive attributes. E-patients are still an underutilized resource. However, embracing e-patients’ ideas and engagement to a higher degree could potentially improve collaboration with health care providers [3,5] as well as quality of care.

### Moving From the First to the Second Generation of E-Patients

Different concepts, sometimes with overlapping definitions, are used in the literature when trying to better understand why patients and informal caregivers actively engage in their self-care and in health care, and how eHealth plays a part. The *highly informed patient* —such as the first generation of e-patients—is described as using the internet to search for information regarding a specific problem and to seek support from peers in online communities [6]. *Expert patients* are described in the early 21st century as patients with chronic conditions who are confident, informed, and knowledgeable and have the skills to take a central role in their self-care and management of care [7]. With a substantial understanding about their condition and context, expert patients have skills in self-care and in working as partners to health care professionals [7,8]. The *digitally engaged patient* is a concept describing that patient engagement encourages the use of digital media technologies for self-care [9]. The concept of a *lead patient* has its roots in the term *lead users* used in design sciences to describe users who face a need before the general market and create their own solutions for this need [10]. The term lead patients is hence used to describe patients or informal caregivers who benefit considerably from finding solutions to their health- or health care–related needs. Lead patients have also been described to contribute to development and important innovations in their self-care and within the health care system [8]. Both digitally engaged and lead patients are examples of the second generation of e-patients. Patients and informal caregivers are also increasingly engaging in the improvement of health care systems. In Sweden today, there are examples of patients employed by health care providers [11]. Their lived experiences and expert knowledge regarding health and care are thereby captured and used to provide a more patient-centered, integrated model of care. Employments of this kind mostly occur within mental health care, for example, the *peer support workers* concept in the United Kingdom [12]. However, our research focuses on a Swedish context. With almost 50% of the Swedish population having chronic conditions [13] and 94% of the population using the internet (with a large variation regarding frequency) [14], there is a great potential for the digitalization of self-care and health care.

### Second Generation of E-Patients—Innovators

Eysenbach and Diepgen described a potential for citizens to manage their own self-care and collaboration with health care with the use of different eHealth solutions [15]. These solutions are suggested to contribute to increased information and control, resulting in more empowered patients. Research suggests that when designing new technological solutions, it is important to have an understanding of the needs of the end user [16,17]. These needs are often connected to the users’ context and personal capabilities and behaviors [18]. Patients’ care experiences may depend on their needs and can be highly individual [19]. Research has also demonstrated that patients are often capable of long-term self-care [20,21] as well as being important contributors to health care development and care delivery [20,22-24]. We see a growing movement of patients and informal caregivers using or creating technological innovations adapted to their specific needs, such as the diabetes patient Dana Lewis who created an artificial pancreas with an open-source approach [25]. They could be described as lead patients or e-patients who have taken their engagement to a new level. However, there is a lack of knowledge about how this second generation of e-patients goes beyond searching for and sharing health information on the Web.

We therefore aimed to describe the second generation of e-patients through exploration of their active engagement in self-care and health care.

## Methods

A qualitative approach with semistructured interviews was used to get a deeper understanding of the second generation of e-patients to describe them.

### Recruitment and Sampling

We purposely recruited participants who could be considered as being part of the second generation of e-patients. The recruitment was performed through convenience and snowball sampling [26]. A Web-based advertisement was published (a website reaching 9054 unique visitors), and through a newsletter (reaching 1500 subscribers). Patients and informal caregivers in Sweden were targeted. Active patients or informal caregivers could either volunteer or be suggested by someone else. A short description of the characteristics of an e-patient was provided (Multimedia Appendix 1). We received 67 suggestions in total, whereof 12 were suggestions from someone else (other patients). A purposeful selection was performed to cover different chronic conditions, different ways of being actively engaged, different locations in Sweden, being a patient or informal caregiver, gender, and age. Overall, 7 out of 15 selected participants were suggested by someone else.

### Data Collection

An interview guide was developed by 3 of the authors (TSD, SR, and MH) based on the literature regarding e-patients. The interview guide consisted of 4 themes: *Background*, *Your health journey*, *Health behavior*, and *Your role in self-care and health care*. The questions were open ended, with the aim of letting the participants talk about what matters most to them. This enabled the researchers to capture narratives that were not foreseen [27]. The interview guide was pilot tested with 5 patients and informal caregivers. The purpose of this pilot testing was to improve the interview guide, and the questions in the guide were slightly altered based on feedback from the pilot interviews. The data from the pilot interviews were not included in the analysis or in the results of the study. Data from the 15 interviews were collected from October to December 2017 through interviews conducted face-to-face (n=6) or over telephone (n=9), depending on the participants’ preferences. The first author and 4 coworkers from a project called “Lead patients”, all with experience of qualitative research interviewing, conducted the interviews. The semistructured interviews took an average of 40 min each. All the interviews were recorded, and the recorded files were transcribed verbatim.

### Data Analysis

The collected data were analyzed according to the framework analysis (FA) approach, with a focus on identifying the occurrence of different concepts regarding e-patients’ active engagement in health and care [28,29]. The analysis process began by producing an initial framework from what was already known to the authors: the second generation of e-patients is highly motivated to be actively engaged in their self-care and in health care. Therefore, we used the self-determination theory (SDT) as a motivational theory and initial framework for our study.

#### Initial Framework

**Figure 1:**
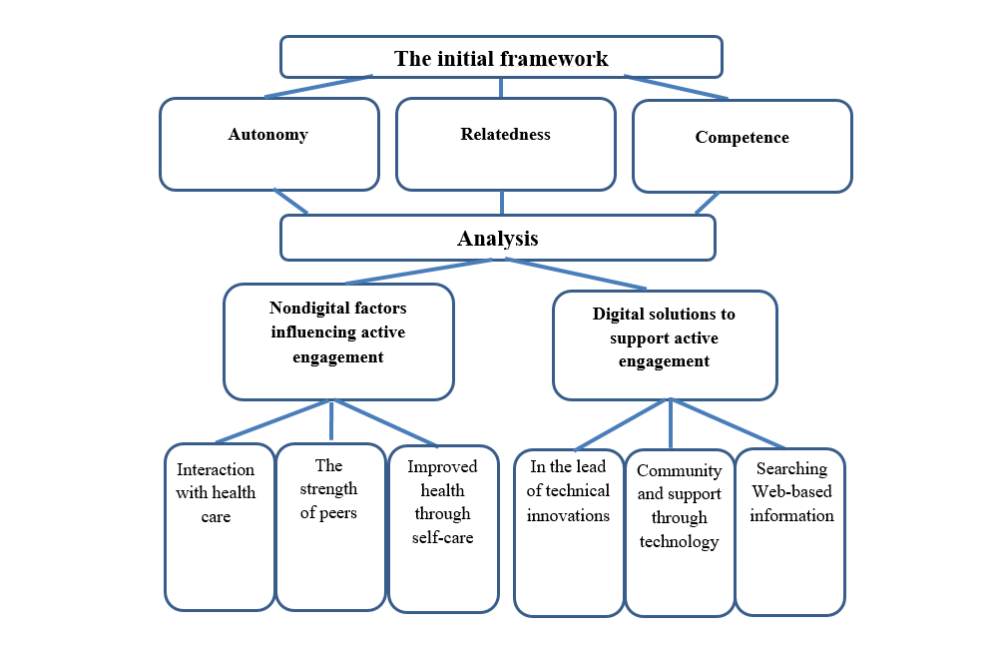
Themes and categories building on the onset of the 3 basic psychological needs from the self-determination theory.

The 3 basic psychological needs from SDT—autonomy, relatedness, and competence—provided a start to describe e-patients’ motivation [30,31] (Figure 1). Using SDT as an initial framework enabled us to explore how participants engage to meet these psychological needs [32]. Autonomy can be described as the capacity and independence to make decisions. Relatedness implies feeling a meaningful connection with others. Competence comprises feeling efficient and able to master difficult situations. The theory argues that these basic needs provide the energy and direction for individuals to act to satisfy their psychological needs, a long-lasting motivation that facilitates persons to value the activity itself and not only an outcome of the activity [30-33].

#### Organizing Data

We followed the 5 steps according to the framework analysis model (familiarization, identifying a thematic framework, coding and indexing, charting, and mapping and interpretation) [28,29] using SDT as an initial framework. As a first step, we familiarized ourselves with the transcribed data, and each interview was given a label. To categorize the content from the interviews, the data were organized into themes, categories, and subcategories (Multimedia Appendix 2). It was an iterative process as we did not want to force the data to fit into the initial framework. The themes were updated multiple times during the analysis process. The meaning, relevance, and importance of the themes were considered and compared with the aim of the study. Sections of data were identified and indexed to match the different themes. All data were indexed, while keeping the links to the specific interview by labels. To ensure that all data were included, color coding was applied to the raw data in the transcribed material. By mapping and interpreting the coding, it was possible to define concepts and find relationships. The beliefs of the participants regarding their reasons for being e-patients and what support they gained from different eHealth solutions were recited [28,29]. To ensure trustworthiness in the analyzed data, at least 2 of the authors were involved at each stage of the analysis. Translation into English was done after analyzing the data, and the translation was verified by all the authors.

### Ethical Considerations

The participating e-patients were provided with written information concerning the purpose of the study and how the researchers would manage their data before signing the informed consent. It was emphasized that participation was voluntary and that it was possible to withdraw at any time without explanations. The requirement of confidentiality was fulfilled. On the basis of a decision from the ethical board in Stockholm, legislation regarding ethical review was not applicable to this study (decision 2015/1572-31/4).

## Results

### Demography and Description of Themes

Semistructured interviews (n=15) were conducted with patients with chronic conditions (n=10) and informal caregivers (n=5). The participants had different diagnoses (Table 1), gender (10 females, 5 males), and age (range 34-77 years; average age per category was as follows: female patients, 50 years; male patients, 61 years; female informal caregivers, 52 years; and male informal caregivers, 64 years).

Subcategories and categories were formed from the data, as described in the Methods section, and 2 overall themes emerged (Figure 1; Multimedia Appendix 2). The theme *nondigital factors influencing active engagement* has 3 categories describing the second generation of e-patients. Their active engagement was influenced by the interaction with health care providers, the strength of peers, and the focus on their own well-being through self-care. The theme *digital solutions to support active engagement* represents the participants’ relationship to eHealth solutions. This theme includes innovations by the second generation of e-patients and how support and learning aspects can be achieved from online communities as well as when finding information on the internet. Within the themes, we found the 3 basic psychological needs from the SDT: autonomy, relatedness, and competence. Going from the initial framework with motivated second generation of e-patients, we found more components to why, and how, they actively engage in their own or others' health and care.

**Table 1:** Demography of participants. Participant identification consists of M: male; F: female; P: patient; and I: informal caregiver.

Participant identification	Type of informal caregiver	Diagnosis
FP1	—^a^	Connective tissue disease
FP2	—	Fatigue syndrome + fibromyalgia
FP3	—	Systemic sclerosis
FP4	—	Mental illness
FP5	—	Irritable bowel syndrome + motility disorder
FP6	—	Parkinson disease
FP7	—	Rheumatic disease
MP1	—	Myocardial infarction
MP2	—	Kidney failure + kidney cancer
MP3	—	Multiple sclerosis
FI1	Mother	Hypersensitivity
FI2	Wife	Thymus neoplasms
FI3	Mother	Down syndrome + heart failure
MI1	Husband	Mental illness
MI2	Husband	Pulmonary fibroses + liver cancer

^a^Missing data, as they are not informal caregivers.

### Nondigital Factors Influencing Active Engagement

Looking into how the participants actively engaged in their self-care and in health care, we found that they strive for an adaptable health care system, wished to collaborate with health care providers (regardless of whether it was primary or specialized care), and wanted to receive feedback regarding their self-care. These 3 aspects were often described as problems needed to be solved. Other important aspects in the patients’ and informal caregivers’ lives were also mentioned: to take a greater responsibility regarding health, to be engaged with peers, to tell their story, and to increase their knowledge. These aspects were often considered as solutions for the problems they experienced. Some participants also described how they strived to be diagnosed correctly and to be believed and taken seriously when in contact with primary health care and the society. All 3 basic psychological needs of SDT (autonomy, relatedness, and competence) were brought up as motivational factors for their active engagement. However, the most consistent factor was to care for others—relatedness. The participants expressed how they wanted to share their experiences with both their peers and the health care professionals. In addition to the 3 SDT needs, all informal caregivers and some patients described how they felt that their motivation to be active was not even a choice.

#### Interaction With Health Care

The participants experienced that practice and knowledge regarding different diseases and conditions was rather low at different health care providers, especially within primary care. The participants expressed that they understood how difficult it may be for health care professionals, regardless of primary or specialized care, to have specific knowledge regarding all diseases. However, these patients meant that they were often confronted with disbelief regarding their specific situation and wished to be approached with more respect regarding their symptoms. Hence, the patients expressed how they needed a more adaptive system that could meet their different requests in a better way. Some of the participants had observed unsafe situations when incorrectly diagnosed and treated both in primary and specialized care, resulting in long-winding situations with misunderstandings. In addition, they sometimes described the health care system as not being able to meet different expectations of engagement from the patients’ side:

The interaction with healthcare is a lot about calling someone on the phone. However, many of the people I’ve met in psychiatry have affective and social difficulties, and for them that is very difficult.

MI1

Most of the participants, during some limited period, experienced lack of support and sustainability in their relationship with primary or specialized health care:

I got a lot of help [from healthcare], but it wasn’t the kind of help that I needed.

FP4

This together with previous experiences of incorrect diagnosis and treatment, motivated a few of the participants to regularly read their medical records online, to ensure correct diagnosis and treatment.

I always log into my health record and check the content. I have also started to record all my conversations in healthcare, to confirm what we actually discussed...

FP5

Being actively engaged was described as a means for the patients to solve health-related problems or misunderstandings at different health care providers. However, it seemed important not to dwell on their own situation or resort to self-pity and apathy. Several participants mentioned how they searched for research regarding their conditions, to increase their knowledge as well as to provide their physician with more information. Participants wanted to share information with their health care professionals as they considered them not having the time to search for new or existing research:

I have read a lot of research, and when I had a physician that was interested in research, we could use that new research to decide on my medication together.

FP3

Some of the participants stressed the importance of making their own judgements and not only listening to the health care professionals. These participants wished that health care professionals could be more constructive by encouraging patients to propose their own ideas and solutions and to give support in this process. To influence the direction of future research, some patients wanted to contribute to new ways of generating research ideas. Therefore, they had chosen to be research partners. However, their agenda was often not met:

The idea was really that I would give input on how the result can be communicated in a way so that patients understand... My own agenda was to see how much influence I can have. Where is the line for how critical I can be as a patient and layman, albeit knowledgeable?

MP1

One mentioned aspect was also to learn from each other, both from health care professionals and from peers, as well as to learn from different situations. Several participants experienced that when their gained knowledge was acknowledged and valued and they felt listened to, they increased their relatedness and continued to be active. Most of the participants expressed that they gained a lot of their knowledge from going through problematic and demanding situations. They would find meaning from these difficulties and challenges as they provided opportunities for learning:

My strategy is to learn from what happens... and as long as I learn from these difficult situations I go through, I just can’t lose.

FI3

Some of the participants described how they are frequently lecturing or teaching in different contexts, with the goal of improving the health care system. With a perspective that comes from a different side of the health care system, they expressed how they gave inspiration and were highlighting new perspectives of existing problems:

My role is to be a catalyst for inspiration and change... I call it an “Accident Investigation Authority.”

MI2

Some patients described that they were working as professional patient peers in specialized clinical contexts. They explained how they assisted other patients in clinical settings, as it could be easier to tell professional peer patients about their needs. One of the patients explained that her role as a patient peer was to encourage others to use different digital solutions for their self-care and to be prepared for their clinical encounter:

When new patients arrive to the clinic, I can help them with their questions, since I have different knowledge and perception than healthcare professionals... That means a lot for the patients.

FP7

Some participants also mentioned the importance of being a patient representative and collaborating with health care providers in quality improvement work:

...it’s a part of how I lead my life, we have to help each other out. That is what keeps me going.

FP3

#### Strength of Peers

Several participants explained how they found inspiration and strength in the relationship with peers through different online communities. This included support on how to navigate the health care system and coordinate their care within different health care contexts or to get suggestions and support for their self-care. The participants were sometimes looking for this support when they experienced insufficient collaboration or interaction with health care professionals or providers.

One patient reasoned about the importance of peer support before an encounter with health care professionals. If several peers had experienced the same problem, it was easier to discuss it with health care professionals, knowing they were not alone regarding their problems legitimized the issue:

...all these patients I talk to in social media... their experiences, reflections and stories are really important. They make me feel less lonely.

FP6

Just as important as finding strength in peers, the participants also found strength in helping others. Several participants found meaning in telling their story to the rest of the world, and by doing so, they indirectly helped other peers. By being peers themselves, the participants stated that they can make a difference with their story.

#### Improved Health Through Self-Care

Several patients expressed that self-care was often part of a life-changing process. They believed that self-care would help and even be necessary in reaching their goals of increasing or maintaining their health:

Healthcare professionals had given up on me before I even entered the room... After that I met a very competent doctor and I asked: What can I do myself? That is the message I have communicated for the last 15 years...

MP2

Both patients and informal caregivers often had concerns for their families, and sometimes being active was described as a result of guilt:

It was a defining moment... I realised that there is actually something I could do to improve my chances of a good life. And I felt that I owe my kids that, at least to give it a try.

MP3

Through extensive experience in self-care, several participants had learned how to recognize signs that needed attention. These signs could be the key for a correct diagnosis or dose of medication or for assessing when acute help is needed. Some participants also described how they learned about their condition by solving a puzzle for their specific situation:

They [health care providers] had answers for each separate issue, but I could see that all these separate issues are somehow connected to each other.

FP3

The informal caregivers described taking on heavy responsibility to ensure good quality of care and self-care for their next of kin. They expressed how they felt that they did not have a choice and that they needed to be in control, otherwise they believed everything would collapse:

I don’t believe they would make it without me... It is always me taking care of health care and school, while my husband is doing things I don’t have time for at home. I’m the one with the whole responsibility.

FI1

To track health, well-being, and medications, some patients described how they used self-tracking to accomplish self-care. Furthermore, some participants indicated that self-care focused on keeping track of their limits and adjusting their daily activities to their current ability. These activities included work, taking care of family, food, physical activity, and other aspects of daily living. If it exceeded their limits, there could be setbacks or relapses. The participants also increased their expert knowledge through research within the field and acted accordingly in relation to lifestyle, for example, diet and physical activity. Several participants had the impression that it is important to be in control of their lives, both in relation to their disease and to life in general:

It’s more about recovering and learning how to deal with your life so that it doesn’t consume you.

FP4

All e-patients explained how they chose not to give up and to use their coping strategies to the best of their ability. To do so, some of them separated their chronic condition from their personality and others went into a role facing the most difficult situations:

For me those are two different things: how I am as a person, my personality, and how my body works and the limitations my disease brings. They are not one and the same.

FP2

However, there were also descriptions of different periods in the participants’ lives when they chose not to be in control of their disease.

### Digital Solutions to Support Active Engagement

Several participants expressed that they need to be able to use different kinds of technical solutions as these solutions played an important role in their self-care, in communication with health care professionals, and when reaching their peers. The participants all used different eHealth solutions or had ideas for new solutions to achieve all of the above.

#### In the Lead of Technical Innovations

All e-patients explained how they saw the potential for future innovations—technical or nontechnical—that would help them and others. Among them, 4 participants expressed ideas of new improvements or had already developed new technical innovations. Several e-patients reported that they were digitally helping peers with their self-care and care coordination or that they were supporting health care providers in their improvement work. In addition, 2 participants had developed their own digital solutions:

I have digitalized a questionnaire for primary care to be able to decide where to send a referral for rare diseases to specialized care, in order for the patients to get the correct diagnosis faster.

FP1

I programmed a web page that I run for my peers... I do it to facilitate for people to share experiences to help each other. I actually have such a close relationship with my doctor that he answers questions from the community on the web page.

MP3

Two informal caregivers had ideas for innovations that could better satisfy their needs. These were models for improved engagement and information exchange between patients, informal caregivers, and health care professionals that displayed and took into account the patients’ daily experience over time:

...I have a great need for an easy method to keep track of side effects. If I had the strength and the energy to do it, I would have created an app for it...

FI2

There are many things to remember since the last encounter, and that is completely hopeless for many people. It’s impossible... I have an idea of using activity trackers for people with mental health issues, to register important aspects of the disease automatically, as an objective measurement...

MI1

#### Community and Support Through Technology

The participants often mentioned social media as a means to find more or less formal communities of peers. In these communities, some participants wrote to inspire or drive for improvements or communicated knowledge gained during their time as patients and informal caregivers. The participants described their use of social media, writing blogs, producing debate articles, writing newspapers and books, and creating a webpage for their peers as support for their activity. Often, their narratives were related to a health care system or societal concern or a problem regarding their health. It could also be aimed at educating peers in self-care:

I have educated many peers through the Internet, so they can answer the most common questions regarding our condition. However, communities still want me to answer more complicated issues... I have become a source of information.

FI1

Other participants explained how they used their knowledge and perceptions to educate others through presentations at different meetings and conferences. However, this is described as physically challenging for some, and they explained how they used video conferences instead to communicate their knowledge:

I’m bedridden six to nine months a year, so technology is crucial for me in order to be active... I use video calls a lot.

FP1

Different types of solutions were used to facilitate self-care, such as devices that track health-related changes, for instance, blood pressure, oxygen uptake, heart rate variability; use of technical support for disabilities; and accessing their electronic health records on the Web.

#### Searching Web-Based Information

Most patients and informal caregivers searched for information on the internet. They were pursuing and finding relevant information all over the world thanks to the internet, which required the participants to learn different languages and understand different contexts. The information could be research or life experiences from peers in different communities or social media. However, the participants explained how they avoided blindly following any information they found, instead they searched for patterns regarding specific health issues. This selective approach was reported by some to be a result of previous negative experiences of being misdiagnosed:

I went home from the health care visit and started googling Fibromyalgia. However, I felt that I didn’t belong there—it was not my diagnosis.

FP3

The participants explained how they found information on the Web that increased their understanding of how the body works. Despite all the support from Web-based communities and information they found when searching the internet, the e-patients reported that they also needed feedback and collaboration with health care professionals regarding this information:

The answer can never be not to google. It has to be: let’s talk about this—how can we relate to this?

FI2

## Discussion

### Principal Findings

This qualitative study contributes to a better understanding of the second generation of e-patients and what they do in their active engagement. The participants engage with others in mutual concerns—family members, peers, and sometimes health care providers. These e-patients describe how they move from problems to finding solutions for their interaction with health care providers and self-care. When interaction with health care providers is perceived as being insufficient, they seek support from their peers. This shows that their relatedness seems to be strong, thanks to the strength of peers, as their collaboration with health care professionals is rather weak. Competence increases through the use of technology and from learning experiences with peers. Their autonomy is important; however, it is sometimes described as involuntary and that it is not really an option to give up. This is because of social contexts and what is expected of them within these contexts, which can be seen as powerful interventions for becoming motivated [32], as the participants sometimes experience that they do not have a choice. There are always different aspects of the social context—the health care system, eHealth solutions, and peers—that will enhance or undermine the individuals’ possibilities to be engaged and active. The relationship between contextual factors and the individuals’ psychological needs will thus affect their well-being and development. One of the factors influencing the participants’ active engagement is striving for an adaptable health care system to support them in their self-care and to be listened to. The use of different eHealth solutions are described as seeking information on the Web, use of apps for managing disabilities or to be involved with health care providers, self-tracking, writing blogs, Web-based access to medical records, social media, or programming their own solutions.

The second generation of e-patients is actively engaged in influencing both their own self-care and the health care system to improve the health of themselves and others. This is done at different levels, such as using their own knowledge and experience and lecturing for health care providers, working as professional peers in clinical settings, and performing self-tracking to give their health care providers a possibility to understand their situation by interpreting the patients’ own data. The first generation of e-patients searched for health information, or resources, on the internet or used online communication with peers [3]. Shaw et al described the use of eHealth such as *health in our hands*, *interacting for health*, and *data enabling health* [21], which lead us to the second generation of e-patients. They still search for health information and communicate health information in different contexts. However, the second generation also generates information themselves, such as described in *health in our hands* [21]. They are enabled to use different digital devices for performing self-tracking, share their knowledge on the Web, have ideas about innovations for solving their needs, and some of the patients have used their programming skills to develop innovations both for their peers and for health care providers. Even though health care is a complex system, and not always suitable for individual solutions, the second generation of e-patients has the knowledge of the system through their lived experience—generally being part of the system for a long period of time. The second generation of e-patients is not only looking for solutions through the internet, this generation is also equipped with providing solutions by sharing information from their own data and creating new interventions for their direct needs. We can compare the evolution of e-patients with that of the World Wide Web, where the Web 2.0 represents the move from static Web pages to dynamic pages with user-generated content and a participatory culture [34]. Similarly, the early e-patient movement has evolved from searching for content and support online to generating knowledge and solutions that are shared. They are engaged, equipped, enabled, and empowered early adopters and innovators that meet their own needs as well as the needs of others. We can see how the participants make purposive choices and act upon these choices in the sense of how the World Bank describes empowerment [35].

The use of digital solutions is one aspect of the social context that might enhance or undermine the possibility to act to satisfy the psychological needs. The results of this study do not provide us with information of whether the use of digital solutions encourages intrinsic motivation or if already perceived motivation lead to an increased use of digital solutions. There are examples from the literature where digital solutions do not increase motivation. Choi et al explored how well a smoking cessation app met the psychological needs and found that it did not encourage autonomous motivation [36]. Still, we argue that the use of digital solutions for self-care should be supported and encouraged by health care providers [37,38] to give a stronger sense of autonomy. This could include guiding patients and informal caregivers within the fragmented health information on the Web [39]. However, building a care relationship can be more or less difficult as actively engaged patients can be challenging for health care professionals [40]. We found that the motivation for the participants varied over time and often depended on context and could therefore not be considered a persistent state. Differences in biology, personality, and individual differences also influence consistency [30]. Threats or opposition could challenge any kind of motivation [41]. Therefore, it is important to have encouragement from both health care professionals and peers [37,38].

### Strengths

The findings of this study provide a broader view of the second generation of e-patients and their active engagement. Most of the 15 participants could be seen as representatives for other digital pioneers within the population of the second generation of e-patients. The concept of e-patients includes informal caregivers as participants. By including informal caregivers, we also obtained views from a group of people often not included in research, and it gave us a broader view by identifying the needs of the severely ill patients that the informal caregivers care for. To learn about the second generation of e-patients and their active engagement enabled us to see how they are slowly transforming the health care system through innovations and being early adopters. Here, we found an interesting interplay between 3 types of actors: the health care system, peers, and the e-patient him or herself.

### Limitations

The result could be affected by the participants’ different chronic conditions as different diseases may give different incentives to actively engage. However, we consider the variety of health issues represented in the study as a strength, and we were able to capture similarities across diagnoses. Regardless of diagnoses, several participants had experienced difficulties in being correctly diagnosed. This could be seen as a need specific for patients with rare diseases; however, our data indicate that it also happened to patients with more common diseases. This was a reason for engaging regardless of condition, such as for patients with mental illness, fibromyalgia, rheumatic disease, cancer, or rare diseases. We did not divide the result between patients and informal caregivers and compared them, even though their needs ought to be different [32]. This is because of the individual differences within the group of informal caregivers as they all had different prerequisites: being a mother to an adult or to a child or being a husband to someone still alive or not. This could however be relevant with another study design, to see the differences within the group of e-patients. There was a rather small group of informal caregivers volunteering (n=5); however, they were all included in the study. Participants were mostly represented by female patients (n=7), which was the dominant group in the recruitment process (n=56). An obvious limitation with a study population of 15 participants is that it is rather challenging to generalize the result to a larger population, even though saturation was reached [19]. Further research is therefore necessary to provide more knowledge about the second generation of e-patients, to further explore individual differences within the concept, and to look into different aspects of possible impact on health care settings. It is also important to see how the health care system, the society, eHealth developers, and research can meet the needs of the second generation of e-patients. This will lead to better solutions, both organizational and digital or technical, for all different kinds of patients and their informal caregivers.

### Conclusions

This study contributes to a better understanding of the second generation of e-patients and how they actively engage in their self-care and in health care. This enabled us to see how they are seeking collaboration with health care professionals and peers and how they interact through innovations. The participants are actively engaged in influencing health care to improve the health care system and their own and others’ health. This indicates that their relatedness seems to be strong. Their competence increases through the use of technology and self-care and from learning experiences with peers and sometimes with health care providers. Their autonomy is sometimes described as involuntary and not possible to give up. It is described as important to have a sufficient collaboration with health care professionals and peers and to have support for their active engagement to continue being motivated and to be an e-patient. Similar to the first generation of e-patients, the participants frequently search for Web-based information. However, the second generation of e-patients also produces their own health data, which they learn from and share, as well as comes up with ideas for digital innovations to meet health-related needs. Taking advantage of the technological development comes naturally for the second generation of e-patients, even if the health care system is not prepared to meet them on these new terms.

## Appendix

Multimedia Appendix 1 The recruitment text.

Multimedia Appendix 2 The categorization process of the themes.

## Abbreviations

eHealth: electronic health
SDT: self-determination theory
