# Supplementary material for: From Information Seekers to Innovators: Qualitative Analysis Describing Experiences of the Second Generation of E-Patients
Source: J Med Internet Res. 2019 Aug 15;21(8):e13022. doi: 10.2196/13022 (PMC6714498; doi:10.2196/13022)
Supplement: Multimedia Appendix 1 [file jmir_v21i8e13022_app1.pdf]

The recruitment text

“Engaged patients or informal caregivers that do more in their self-care and health care, than expected of them. E.g. searching for health information online, have ideas of new solutions/innovations for their self-care and in communication with health care, or track their health to gain further knowledge.”
