# Supplementary material for: From Information Seekers to Innovators: Qualitative Analysis Describing Experiences of the Second Generation of E-Patients
Source: J Med Internet Res. 2019 Aug 15;21(8):e13022. doi: 10.2196/13022 (PMC6714498; doi:10.2196/13022)
Supplement: Multimedia Appendix 2 [file jmir_v21i8e13022_app2.pdf]

Multimedia Appendix 2. The categorization process of the themes.

Sub-categories	Categories	Theme
Striving for a change	Interaction with health care	Nondigital factors influencing active engagement
Patient safety		
To gain support for health and care		
Patients that lead the way	The strength of peers	
Not being alone		
Help me navigate and coordinate the care		
To monitor and measure over time	Improved health through self-care	
Self-care as healing and preventive		
To make changes in my way of living		
Moving from manual to digital systems	In the lead of technical innovations	Digital solutions to support active engagement
Pursues the digitalisation		
Digital services that facilitate		
Social media	Community and support through technology	
Webinars		
Web-based support		
Web-based research	Searching Web-based information	
Searching engines		
Finding patterns		
Newsletters		
